# Pan-Soft Tissue Sarcoma Analysis of the Incidence, Survival, and Metastasis: A Population-Based Study Focusing on Distant Metastasis and Lymph Node Metastasis

**DOI:** 10.3389/fonc.2022.890040

**Published:** 2022-07-07

**Authors:** Haotian Liu, Hongliang Zhang, Chao Zhang, Zhichao Liao, Ting Li, Tielong Yang, Gengpu Zhang, Jilong Yang

**Affiliations:** ^1^ Department of Bone and Soft Tissue Tumor, Tianjin Medical University Cancer Institute and Hospital, Tianjin, China; ^2^ National Clinical Research Center for Cancer, Key Laboratory of Cancer Prevention and Therapy, Tianjin’s Clinical Research Center for Cancer, Tianjin Medical University Cancer Institute and Hospital, Tianjin, China; ^3^ Orthopedic Surgery Department, Tianjin Hospital, Tianjin University, Tianjin, China

**Keywords:** soft tissue sarcoma, incidence, survival, distant metastasis, lymph node metastasis

## Abstract

**Background:**

The rarity and complexity of soft tissue sarcoma (STS) make it a challenge to determine the incidence, survival, and metastasis rates. In addition, the clinicopathological risk factors for lymph node metastasis have rarely been reported.

**Methods:**

Data on patients diagnosed with STS in the SEER database from 2000 to 2018 were extracted by SEER*Stat 8.3.9.1, and the incidence trend was calculated by Joinpoint 4.9 software. The KM method was used to calculate the survival curve, and the log-rank method was used to compare differences in the survival curves. The clinicopathological risk factors for lymph node metastasis were screened by logistic regression.

**Results:**

Among the 35987 patients, 4299 patients (11.9%) had distant metastasis. The overall lymph node metastasis rate was 6.02%, which included patients suffering from both lymph node and distant metastasis. Considering that some lymph node metastases might be accompanying events of distant metastasis, the rate of only lymph node metastasis in STS patients decreased to 3.42% after excluding patients with distant metastasis. Patients with only lymph node metastases (N1/2M0) had a significantly worse prognosis than those without metastases (N0M0) but a better prognosis than those with only distant metastases (N0M1) (p<0.0001). In the multivariate logistic analysis, STS patients with larger tumors located in the head and neck, viscera, retroperitoneum, and certain specific pathological subtypes (compared with the liposarcoma), such as undifferentiated pleomorphic sarcoma, rhabdomyosarcoma, endometrial stromal sarcoma, gastrointestinal stromal tumor, synovial sarcoma, and angiosarcoma, had a higher risk of lymph node metastasis.

**Conclusions:**

Lymph node metastasis is rare in STS, and the metastasis rate is significantly different among the different pathological types. Tumor size, location, and pathological subtype are significantly associated with the risk of lymph node metastasis. The overall survival of patients with lymph node metastasis is better than that of patients with distant metastasis, which suggests a more precise prognosis evaluation should be performed in these AJCC stage IV STS patients.

## Introduction

Soft tissue sarcomas (STSs) are a group of highly malignant mesenchymal tumors that can occur at almost any anatomical site, accounting for 1% of all malignant tumors in adults and 15% of all malignant tumors in children (1–3). The incidence of STS varies in different countries and regions, with a crude incidence of 4.7 per 100000 in Europe and 2.91 per 100000 in China (1, 4). Patients with STS have a poor prognosis, with a 5-year disease-specific survival rate of only 50%-70% (5). In contrast to other cancers, the pathological types of STS are complex. It is estimated that there are more than 50 pathological subtypes of STS, each of which exhibits slight differences in their biological behavior and related treatment modalities (6). The low incidence and diverse pathological subtypes make it a challenge to describe the epidemiological characteristics of STS, such as the incidence trend, age at diagnosis, prognosis and metastasis risk.

Lymph node metastases are rare in sarcoma compared to blood metastases, with only approximately 2%-10% of patients having lymph node metastases (7, 8). Risk factors for lymph node metastasis have rarely been described, and only a few studies have reported a high proportion of lymph node metastasis in certain subtypes, such as rhabdomyosarcoma, synovial sarcoma, and angiosarcoma (9–13). However, these studies confirmed the risk of lymph node metastasis using only the proportion of lymph node metastasis, without consideration of the biological features and clinicopathological characteristics of STS. Moreover, some studies were single-institution studies with relatively few patients. Therefore, a large sample study that combines the clinicopathological features of patients is needed to further evaluate the risk factors for sarcoma lymph node metastasis.

The SEER database, which represents 28% of the U.S. population, included 9 cancer registries in 1974, which was increased to 13 cancer registries in 1992 and 18 registries in 2000 (14, 15). The large number of patients and rich variable information help to compensate for the deficiency of single-center data and make the SEER database a powerful tool to study the epidemiology and prognosis of cancer patients.

In this study, by extracting information on patients with STS from the SEER database, we provided statistics on the overall incidence, age at diagnosis, survival, and presence of distant metastasis and lymph node metastasis in patients with STS. Most importantly, we show that the pure lymph node metastasis rate is approximately 3.42% in STS, and patients with only lymph node metastasis have a better overall survival than those with distant metastasis, which suggests that a more precise prognosis evaluation for these AJCC stage IV patients, as well as the identification of risk factors for lymph node metastasis should be performed.

## Materials and Methods

### Study Population

**Figure 1:**
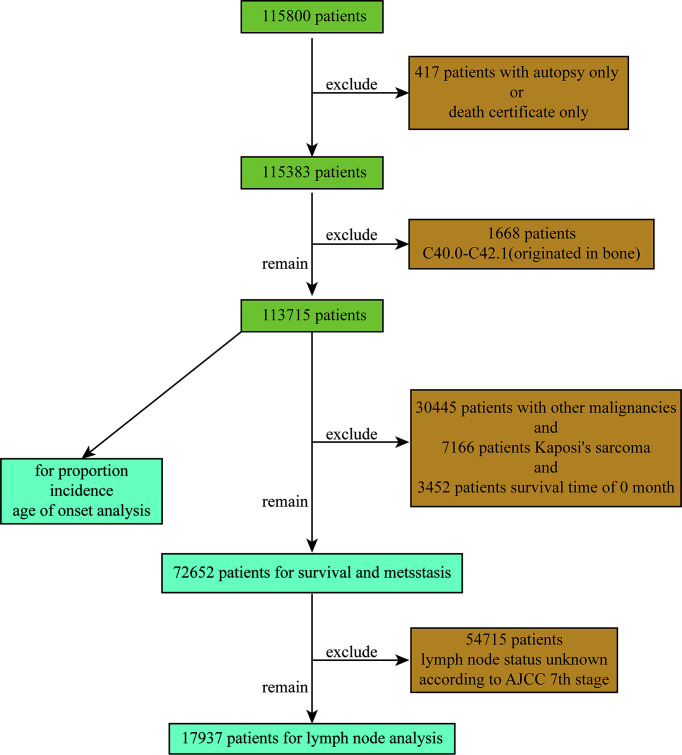
Flow chart used to screen patients.

Based on the ICD-O-3 code, we extracted data on patients diagnosed with STS between 2000 and 2018 from 18 cancer registries in the SEER database. The extracted variables included sex, site, race, year of diagnosis, pathological diagnosis, age, tumor grade, tumor size, AJCC 7th TNM stage, survival status, survival time, type of reporting source, etc. Exclusion criteria included STS confirmed only by autopsy or death certificate and patients with site codes C40.0 to C42.1 (primary in bone). The flow chart used to screen patients is shown in **Figure 1**. A total of 115,800 patients were retrieved, and a total of 113,715 patients were included in the final analysis after excluding 417 patients with only autopsy or death certificates and 1668 patients with primary bone origin. In addition, STSs of similar tissue origin were grouped, as shown in **Supplementary Table 1**. The SEER database is a public open access database, so this study did not require ethics committee approval.

### Statistical Analysis

Age-adjusted incidence was calculated using SEER*Stat 8.3.9.1, based on the 2000 U.S. population, and then the incidence trend and annual percentage change (APC) were calculated using Joinpoint 4.9 software. Age was regarded as a continuous variable in the age of onset. In multivariate logistic analysis, sex, site, race, age, tumor grade, size and tumor subtype were classified as categorical variables. Among them, age was divided into two groups: ≤50 years old and >50 years old, and tumor diameter was divided into three groups: ≤5 cm, 5-10 cm and >10 cm.

The Kaplan–Meier (KM) method was used to calculate the survival curve, and the log-rank method was used to compare the differences in the survival curves. Logistic regression was used to screen risk factors for lymph node metastasis, and the results are expressed as OR values and 95% confidence intervals. All p values are bilateral, and a p value <0.05 was considered statistically significant. All analyses were performed using SPSS 22.0.

## Results

### Proportion, Incidence and Age

**Figure 2:**
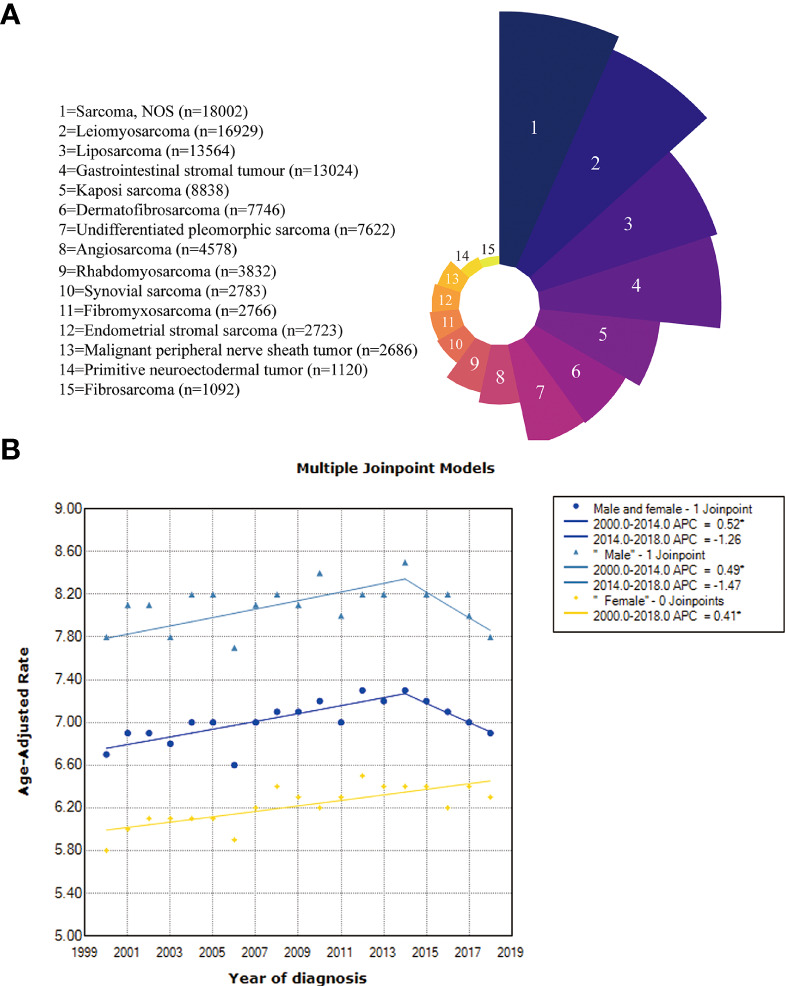
**(A)** The proportion of the pathological subtypes with more than 1000 cases; **(B)** Incidence trends in the overall, male and female patients.

First, we quantified the proportion of patients with each pathological subtype. Perhaps because of the limited diagnosis and treatment methods, the specific pathological subtype could not be determined for a considerable number of sarcoma patients (sarcoma NOS, n=18002, 15.8%). Leiomyosarcoma (n= 16929, 14.9%), liposarcoma (n= 13564, 11.9%), gastrointestinal stromal tumor (n= 13024, 11.5%), Kaposi’s sarcoma (n= 8838, 7.8%), dermatofibrosarcoma (n=7746, 6.8%), and undifferentiated pleomorphic sarcoma (n= 7622, 6.7%) were the most common subtypes (**Figure 2A**).

Then, we calculated the incidence of all patients, males and females (**Figure 2B**). The incidence in the total population increased from 6.7 per 100000 in 2000 to 7.3 per 100000 in 2014 (APC=0.52%, 95% CI: 0.3%-0.8%, p =0.001) but then decreased between the years 2014 and 2018, with no significant difference observed (p=0.13). The incidence of males increased from 7.8 per 100000 in 2000 to 8.5 per 100000 in 2014 (APC=0.49%, 95% CI: 0.2%-0.8%, p =0.008) but also decreased between the years 2014 and 2018, with no significant difference (p =0.088). The incidence trend was slightly different for females, showing an increasing trend from 2000 (5.8/100000) to 2018 (6.3/100000) (APC=0.41%, 95% CI: 0.2-0.6%, p <0.001).

**Figure 3:**
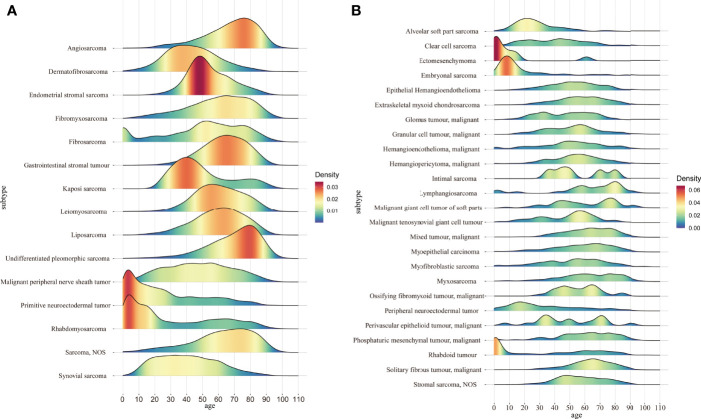
**(A)** Age distribution of the pathological subtypes with more than 1000 cases; **(B)** Age distribution of the pathological subtypes with fewer than 1000 cases.

We next proceeded to determine statistical trends in the age at diagnosis for the different pathological subtypes. Among the pathological subtypes with more than 1000 cases, primitive neuroectodermal tumor and rhabdomyosarcoma were more likely to occur in children and adolescents, while the other pathological subtypes were more likely to occur in middle aged and elderly individuals, with angiosarcoma and undifferentiated pleomorphic sarcoma having the most advanced incidence peak (**Figure 3A**). In the pathological subtypes with <1000 cases, ectomesenchymoma, embryonal sarcoma and rhabdoid tumor were more likely to occur in children and adolescents (**Figure 3B**).

### Survival

A total of 72,652 patients were included in the survival analysis after excluding patients with other malignancies (to avoid the effect of other malignancies on survival and metastasis), Kaposi’s sarcoma (could not confirm whether it is related to HIV infection and avoid the effect of immune deficiency on survival and metastasis), and patients with a survival time of 0 months.

**Figure 4:**
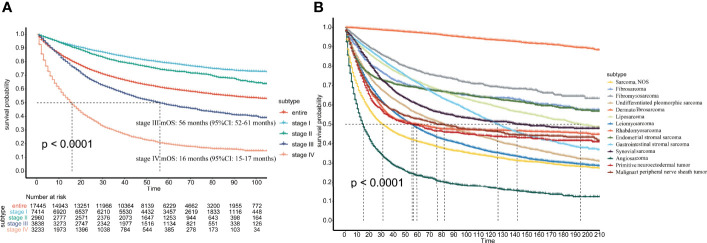
**(A)** Survival of patients with different AJCC stages; **(B)** Survival of patients with different pathological subtypes.

In terms of AJCC stage, there were significant differences in prognosis among patients with different stages. Stage I patients had the best prognosis, and stage IV patients had the worst prognosis. The mOS for stage III and stage IV patients was 56 and 16 months, respectively, and mOS was not achieved in all patients, stage I patients, or stage II patients (**Figure 4A**).

In the total population, the 1-, 3-, 5-, 7-, 10-, and 15-year survival rates were 74%, 63%, 57%, 53%, 48%, and 43%, respectively. For the different pathological subtypes, dermatofibrosarcoma had the best prognosis among the most common pathological types, with 1-year, 3-year and 5-year survival rates up to 99%, 98% and 97%, respectively. Angiosarcoma had the worst prognosis, with 1-, 3-, and 5-year survival rates of 39%, 27%, and 22%, respectively (**Figure 4B**). The 1-year, 3-year, 5-year, 7-year, 10-year, and 15-year survival rates of the other pathological subtypes are shown in **Supplementary Table 2**.

### Distant Metastasis

The distant metastases recorded in the SEER database were located in the bone, brain, liver, and lung. Of 72,652 patients, a total of 35,987 patients were included in the distant metastasis-related analysis after 36,665 patients with unknown distant metastatic status were excluded. Among the 35987 patients, 4299 patients (11.9%) had distant metastasis. Alveolar soft part sarcoma, epithelial hemangioendothelioma, rhabdoid tumor, and rhabdomyosarcoma had the four highest distant metastasis rates (49.57%, 35.92%, 28.99%, and 23.91%, respectively) (**Table 1**).

**Table 1:** Distant metastases rate in different pathological subtypes.

Subtype	No	Yes	Total	Percentage
Alveolar soft part sarcoma	59	58	117	49.57%
Epithelial Hemangioendothelioma	91	51	142	35.92%
Rhabdoid tumour	120	49	169	28.99%
Rhabdomyosarcoma	1120	352	1472	23.91%
Hemangioendothelioma, malignant	29	8	37	21.62%
Angiosarcoma	865	218	1083	20.13%
Phosphaturic mesenchymal tumour, malignant	8	2	10	20.00%
Peripheral neuroectodermal tumor	135	31	166	18.67%
Leiomyosarcoma	4749	1082	5831	18.56%
Sarcoma, NOS	5179	1075	6254	17.19%
(epithelioid sarcoma)	141	30	171	17.54%
Extraskeletal myxoid chondrosarcoma	139	25	164	15.24%
Clear cell sarcoma	99	16	115	13.91%
Synovial sarcoma	952	142	1094	12.98%
Primitive neuroectodermal tumor, NOS	255	34	289	11.76%
Granular cell tumour, malignant	30	4	34	11.76%
Gastrointestinal stromal tumour	4492	575	5067	11.35%
Malignant peripheral nerve sheath tumor	691	83	774	10.72%
Endometrial stromal sarcoma	925	110	1035	10.63%
Embryonal sarcoma	48	5	53	9.43%
Perivascular epithelioid tumour, malignant	10	1	11	9.09%
Stromal sarcoma, NOS	137	12	149	8.05%
Solitary fibrous tumour, malignant	244	20	264	7.58%
Fibrosarcoma	238	19	257	7.39%
Mixed tumour, malignant	102	7	109	6.42%
Malignant giant cell tumor of soft parts	15	1	16	6.25%
Hemangiopericytoma, malignant	181	11	192	5.73%
Myxosarcoma	153	9	162	5.56%
Malignant tenosynovial giant cell tumour	20	1	21	4.76%
Undifferentiated pleomorphic sarcoma	1200	59	1259	4.69%
Myofibroblastic sarcoma	49	2	51	3.92%
Liposarcoma	4915	190	5105	3.72%
Glomus tumour, malignant	26	1	27	3.70%
Myoepithelial carcinoma	235	8	243	3.29%
Fibromyxosarcoma	1220	34	1254	2.71%
Dermatofibrosarcoma	2930	4	2934	0.14%
Ossifying fibromyxoid tumour, malignant	20	0	20	0.00%
Ectomesenchymoma	5	0	5	0.00%
Lymphangiosarcoma	2	0	2	0.00%

First, in terms of metastatic sites, among single-site metastases, the most common site was the lung (n=1768, 4.91%), followed by the liver (n=908, 2.52%), bone (n=453, 1.26%), and brain (n=61, 0.17%). When considering possible combinations of other metastatic sites, the most common site remained the lung (n=2744, 7.62%), followed by the liver (n=1571, 4.37%), bone (n=1160, 3.22%), and brain (n=205, 0.57%).

Second, we calculated the metastasis rates of the different pathological types at four distant metastasis locations: bone, brain, liver and lung. Among the pathological subtypes with bone metastasis, rhabdomyosarcoma, malignant hemangioendothelioma and alveolar soft part sarcoma had the three highest bone metastasis rates (13.59%, 10.81% and 10.26%, respectively) (**Supplementary Table 3**). Among the pathological subtypes with brain metastasis, alveolar soft part sarcoma, rhabdoid tumor, and primitive neuroectodermal tumor had the three highest brain metastasis rates (5.98%, 4.73%, and 3.11%, respectively) (**Supplementary Table 4**). Among the pathological subtypes with liver metastasis, epithelial hemangioendothelioma, gastrointestinal stromal tumor and leiomyosarcoma had the three highest liver metastasis rates (12.68%, 10.8% and 7.22%, respectively) (**Supplementary Table 5**). Among the pathological subtypes with lung metastasis, alveolar soft part sarcoma, epithelial hemangioendothelioma and malignant phosphaturic mesenchymal tumor had the three highest lung metastasis rates (47.01%, 27.46% and 20%, respectively) (**Supplementary Table 6**).

**Figure 5:**
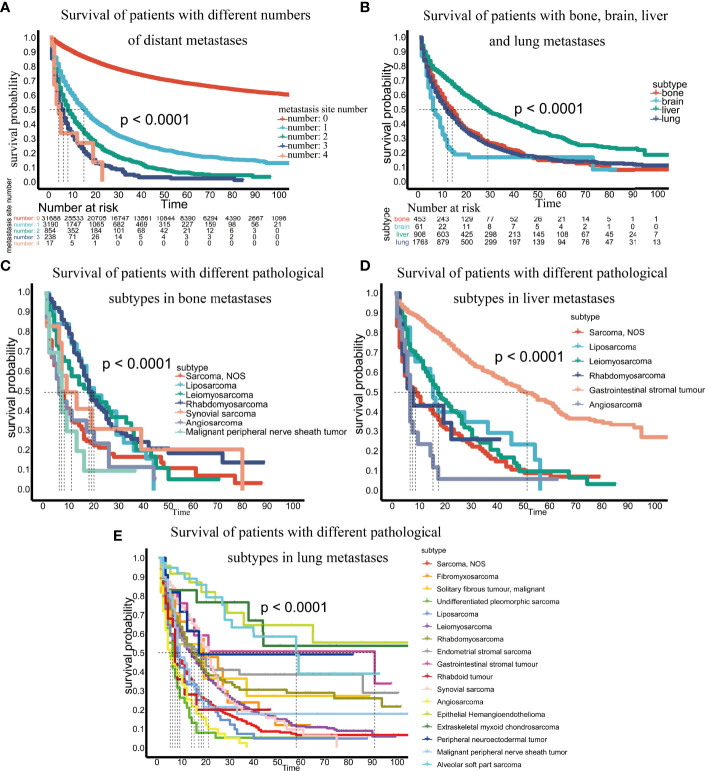
**(A)** Survival of patients with different numbers of distant metastases; **(B)** Survival of patients with bone, brain, liver and lung metastases; **(C)** Survival of patients with different pathological subtypes of bone metastases; **(D)** Survival of patients with different pathological subtypes of liver metastases; **(E)** Survival of patients with different pathological subtypes of lung metastases.

Third, we evaluated the impact of the number of metastatic sites on patient survival and found that more metastatic sites resulted in worse patient survival (**Figure 5A**). We compared the survival differences of patients with metastases at different sites and found that patients with brain metastasis had the worst survival, while those with liver metastasis had the best survival (**Figure 5B**).

Finally, we analyzed survival differences among different pathological subtypes at the same metastatic site. For bone metastases, we analyzed the survival of pathological subtypes with 10 or more patients and found that malignant peripheral nerve sheath tumors had the worst prognosis (p <0.0001) (**Figure 5C**). In brain metastases, the number of patients per pathological subtype was too small to perform survival analysis. In liver metastases, among the pathological subtypes with 10 or more patients, angiosarcoma had the worst prognosis, and gastrointestinal stromal tumors had the best prognosis (p<0.0001) (**Figure 5D**). In terms of lung metastasis, angiosarcoma and malignant peripheral nerve sheath tumors had the worst prognosis among pathological subtypes with 10 or more patients (p <0.0001) (**Figure 5E**).

### Lymph Node Metastasis

Of the 72652 patients included in the survival analysis, 54715 patients with unknown lymph node status were excluded based on the AJCC 7th edition, and the remaining 17937 patients were included in the lymph node analysis.

**Figure 6:**
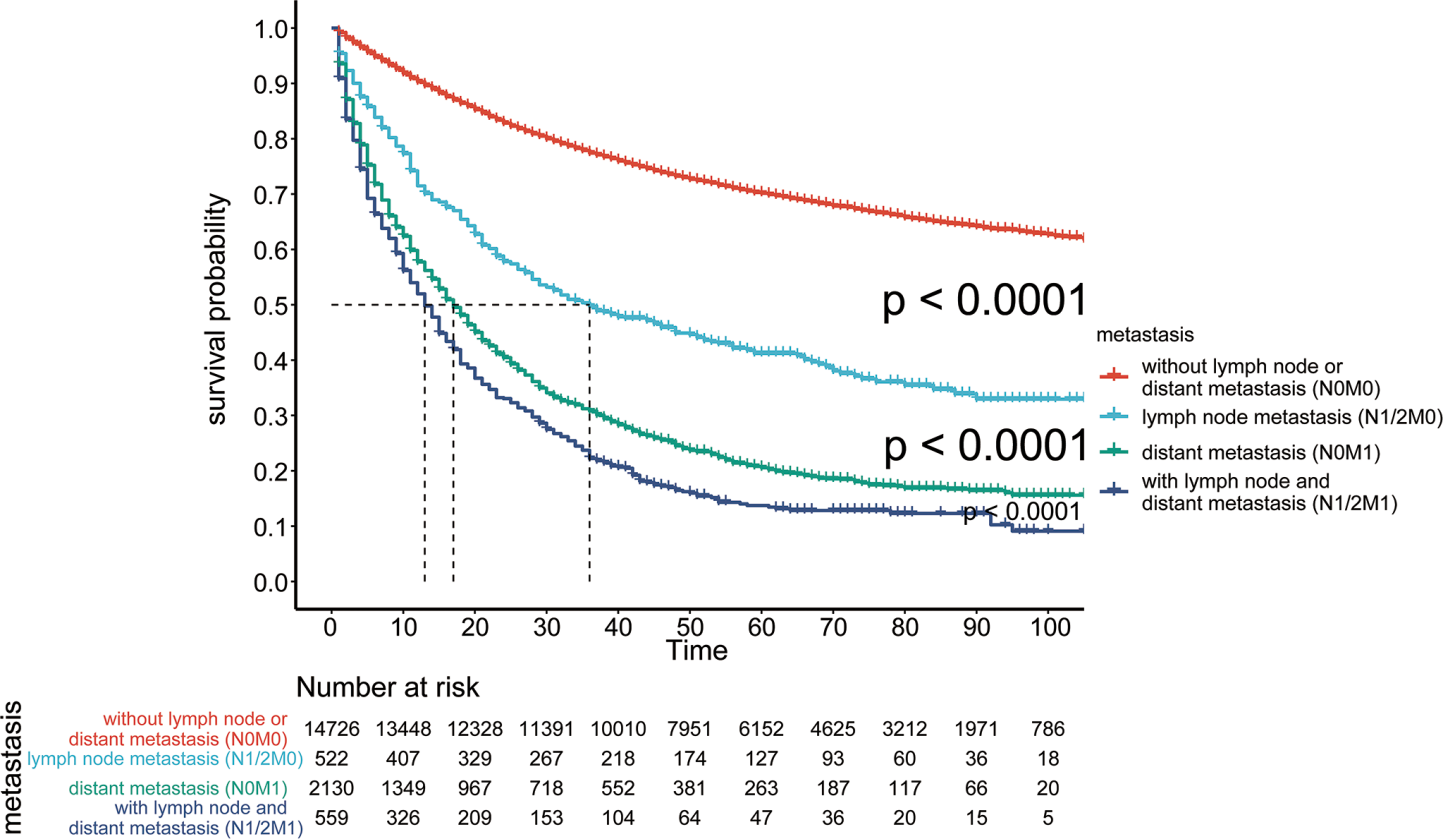
Survival of patients without lymph node or distant metastasis (N0M0), lymph node metastasis (N1/2M0), distant metastasis (N0M1), and lymph node and distant metastasis (N1/2M1).

First, we compared differences in survival among patients with no lymph node metastasis or distant metastasis (N0M0), only lymph node metastasis (N1/2M0), only distant metastasis (N0M1), both lymph node metastasis and distant metastasis (N1/2M1). The results showed that the prognosis of patients with only lymph node metastasis (N1/2M0) was significantly worse than that of patients with no lymph node metastasis or distant metastasis (N0M0) (p <0.0001) and was significantly better than that of patients with only distant metastasis (N0M1) (p <0.0001) (**Figure 6**). The overall 3-year and 5-year survival rates of these three types of patients were 46% and 38%, 74% vs. 68%, and 25% vs. 18%, respectively, suggesting that patients with lymph node metastasis (N1/2M0) and distant metastasis (N0M1) have different prognoses.

Second, we calculated the proportion of patients with lymph node metastasis across the different pathological subtypes. Of the 17937 patients, 1081 (6.02%) had lymph node metastasis. After excluding 2698 patients with distant metastasis (NXM1), 522 of the remaining 15248 patients, accounting for 3.42%, had lymph node metastasis. Among the 17937 patients, given the influence of the number of patients, the pathological types with >100 cases and <100 cases were counted separately. Among the pathological subtypes with >100 patients, the ten most common subtypes exhibiting lymph node metastases were rhabdomyosarcoma (26.88%), angiosarcoma (15.43%), sarcoma NOS (9.39%), endometrial stromal sarcoma (8.51%), myoepithelial carcinoma (6.67%), synovial sarcoma (5.23%), leiomyosarcoma (5.08%), gastrointestinal stromal tumor (4.16%), malignant peripheral nerve sheath tumor (3.13%), and undifferentiated pleomorphic sarcoma (3.03%); the lymph node metastasis rate of dermatofibrosarcoma was 0% (**Table 2**). Among the pathological subtypes with <100 patient cases, the three pathological types with the highest lymph node metastasis rates were malignant phosphaturic mesenchymal tumors (42.86%), ectomesenchymomas (33.33%), and malignant mixed tumors (32.86%), but because the number of cases of malignant phosphaturic mesenchymal tumors and ectomesenchymomas was small (7 and 3 cases, respectively), the statistical efficacy was limited (**Supplementary Table 7**).

**Table 2:** Lymph node metastasis rate in pathological subtypes with patients >100 cases.

subtype	Negative	positive	total	percentage
Rhabdomyosarcoma	468	172	640	26.88%
Angiosarcoma	318	58	376	15.43%
Sarcoma, NOS	2702	280	2982	9.39%
Endometrial stromal sarcoma	602	56	658	8.51%
Myoepithelial carcinoma	98	7	105	6.67%
Synovial sarcoma	598	33	631	5.23%
Leiomyosarcoma	2912	156	3068	5.08%
Gastrointestinal stromal tumor	3040	132	3172	4.16%
Malignant peripheral nerve sheath tumor	434	14	448	3.13%
Undifferentiated pleomorphic sarcoma	544	17	561	3.03%
Fibrosarcoma	144	4	148	2.70%
Liposarcoma	2890	43	2933	1.47%
Fibromyxosarcoma	730	7	737	0.95%
Dermatofibrosarcoma	557	0	557	0.00%

**Figure 7:**
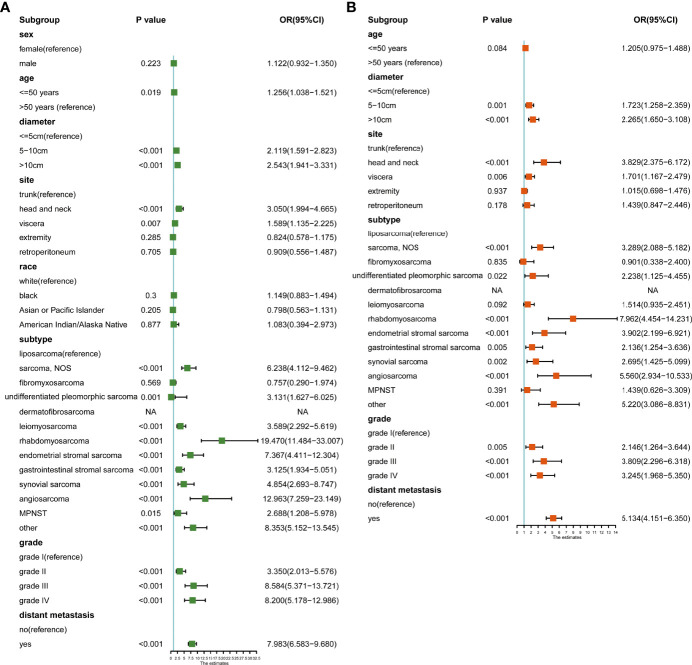
**(A)** Univariate logistic analysis of risk factors for lymph node metastasis; **(B)** Multivariate logistic analysis of risk factors for lymph node metastasis.

Third, we calculated the risk factors for lymph node metastasis. In univariate logistic analysis, age, tumor diameter, site, subtype, grade, and distant metastasis were associated with lymph node metastasis (**Figure 7A**). In multivariate logistic analysis, age was not associated with lymph node metastasis. Patients with tumor diameters of 5-10 cm (OR: 1.723, 95% CI: 1.258-2.359, p=0.001) and >10 cm (OR: 2.265, 95% CI: 1.650-3.108, p<0.001) had a higher risk of lymph node metastasis. Compared to trunk sarcoma, head and neck (OR: 3.829, 95% CI: 2.375-6.172, p<0.001) and visceral (OR: 1.701, 95% CI: 1.167-2.479, p=0.006) sarcoma was associated with a higher risk of metastasis. Compared with the liposarcoma, sarcoma NOS (OR: 3.289, 95% CI: 2.088-5.182, p<0.001), undifferentiated pleomorphic sarcoma (OR: 2.238, 95% CI: 1.125-4.455, p=0.022), rhabdomyosarcoma (OR: 7.962, 95% CI: 4.454-14.231, p<0.001), endometrial stromal sarcoma (OR: 3.902, 95% CI: 2.199-6.921, p<0.001), gastrointestinal stromal tumor (OR: 2.136, 95% CI: 1.254-3.636, p=0.005), synovial sarcoma (OR: 2.695, 95% CI: 1.425-5.099, p=0.002), and angiosarcoma (OR: 5.560, 95% CI: 2.934-10.533, p<0.001) had a greater lymph node metastasis risk. Grade II (OR: 2.146, 95% CI: 1.264-3.644, p=0.005), grade III (OR: 3.809, 95% CI: 2.296-6.318, p<0.001) and grade IV (OR: 3.245, 95% CI: 1.968-5.350, p<0.001) were associated with a higher risk of metastasis. Patients with distant metastasis (OR: 5.134, 95% CI: 4.151-6.350, p<0.001) had a higher risk of lymph node metastasis (**Figure 7B**).

## Discussion

As a rare and highly malignant tumor, STS accounts for more than 80% of all sarcoma subtypes and has a poor prognosis, with a 5-year survival rate of only 50%-70% (5, 16). For patients with locally advanced or metastatic STS, the prognosis is worse, with a median overall survival of 12.8 to 14.3 months (17). Therefore, it is necessary to evaluate its epidemiology and prognosis. At the same time, due to its relative rarity, it is difficult for single-institution data to accurately and comprehensively describe its incidence, survival and metastasis rates, while the SEER database with huge case resources only makes up for the deficiency of data from a single center. In this study, using the SEER database, we briefly outlined the age of onset and trends in STS patients, compared the survival and calculated specific survival rates of different pathological subtypes, and also compared the incidence and survival of different metastatic sites and survival of different pathological subtypes within the same metastatic site. Most importantly, we found that patients with lymph node metastasis alone (N1/2M0) and distant metastasis (N0M1) had a different prognosis (p<0.001), focusing on the percentage of positive lymph nodes for different pathological subtypes and the clinicopathological risk factors for lymph node metastasis, thus providing guidance for STS management.

In the current study, the incidence of STS showed an increasing and statistically significant trend from 2000-2014 in the total population and males and a decreasing trend from 2014-2018 with no statistically significant trend. In females, there was a significant increasing trend from 2000-2014. The different trends in the incidence of STS between males and females suggest that there may be sex differences in the incidence of STS, which needs to be confirmed by further studies. The increased incidence may be due to advances in testing for STS, while the reasons for the decline in the overall population and men in 2014-2018 remain unclear and require further study. In addition, the crude incidence of STS was 2.91/100000 in China (2.72/100000 in males and 3.11/100000 in females) and 4.7/100000 in Europe (1, 4). Compared with other countries, therefore, it can be seen that the incidence of STS is slightly higher in the United States.

As an important part of epidemiology, age of onset plays an important role in the diagnosis and prognosis of patients with STS. The present study is the first to depict the landscape of the age of onset for STS. The results showed that rhabdomyosarcoma and primitive neuroectodermal tumors were more likely to occur in children and adolescents, while the incidence peaks of angiosarcoma and undifferentiated pleomorphic sarcoma were concentrated in middle-aged and elderly people, which provided a basis for the screening and prevention of STS.

Kaposi’s sarcoma can be divided into four main types: classic Kaposi’s sarcoma, endemic African Kaposi’s sarcoma, iatrogenic immunosuppressive Kaposi’s sarcoma and AIDS-Kaposi’s sarcoma, with AIDS-Kaposi’s sarcoma being the majority (18, 19). The SEER database does not provide a specific classification of patients with Kaposi’s sarcoma; therefore, to eliminate the influence of other malignancies and immune deficiency on survival and metastasis, the present study excluded patients with other malignancies and Kaposi’s sarcoma for survival and metastasis correlation analysis.

Although previous studies have also reported survival differences among different pathological subtypes of STS, we not only calculated the survival rates of different pathological subtypes in different years, but also calculated the incidence of different metastatic sites. We also compared the survival differences among patients with different metastatic sites and the survival differences among patients with different pathological subtypes in the same metastatic site (20). Lung metastasis was found to be the most common site of STS metastasis, followed by liver, bone, and brain metastasis, whether single site metastases or possibly combination of other metastatic sites. In addition, among the four types of lung, bone, liver and brain metastasis, patients with brain metastases have a worse prognosis than those with liver metastases, possibly because of the strong compensatory ability of the liver. Even after liver metastasis occurs, there are still some liver cells that can compensatively maintain function to the greatest extent possible, while the brain metastasis often significantly affects the treatment efficacy and life quality. The prognosis of patients with liver metastasis is better than that of patients with lung metastasis, which may be due to the strong compensatory capacity of liver, another reason might due to the different pathological subtypes in the liver metastatic patients compared to lung, bone and brain metastatic patients, because different pathologic type had different prognosis. For an example, the pathological types with poor prognosis included angiosarcoma, sarcoma NOS, leiomyosarcoma, and undifferentiated pleomorphic sarcoma and so on. Among the 908 patients with liver metastases alone, these four pathological subtypes were found in 17 (1.9%), 115 (12.7%), 170 (18.7%), and 2 (0.2%) patients, respectively, for a total of 33.5%. While among the 1768 patients with pulmonary metastases, these four pathological subtypes were found in 75 (4.2%), 528 (29.9%), 506 (28.6%), and 38 (2.1%) patients, respectively, for a total of 64.8%. It can be seen that the total proportion of these four pathological subtypes in patients with liver metastasis was significantly lower than that in patients with lung metastasis. Therefore, we speculated that these two points may explain why patients with liver metastasis have a better prognosis than those with lung metastasis. In addition, malignant peripheral nerve sheath tumors have the worst prognosis in bone metastases, angiosarcoma in liver metastases, malignant peripheral nerve sheath tumors and angiosarcoma in lung metastases. This provides guidance for evaluating the prognosis of patients with STS.

It has been confirmed that the prognosis of patients with lymph node metastasis is significantly worse than that of patients without lymph node metastasis (7). However, some of these studies either included fewer cases because they were single-center studies or did not specify whether patients with distant metastases were included, which may result in bias and affect the accuracy of the results (10, 12, 21, 22). In the present study, we performed three-aspect analysis of lymph node metastasis in STS. First, there was a significant survival difference between patients with lymph node metastasis alone and those with distant metastasis alone (p <0.0001). David et al. also found that patients with lymph node metastasis alone had better outcomes than those with distant metastasis (p =0.0009) and worse outcomes than those without metastasis (p <0.001) (7). Atalay et al. came to similar conclusions (13). Riad et al. further found that there was no significant survival difference between patients with lymph node metastasis alone and patients with stage III STS, although some patients with lymph node metastasis underwent lymph node resection (21). In the AJCC 8th stage, both lymph node metastasis and distant metastasis are classified as stage IV, with poor prognosis. Therefore, we believe that the separation of lymph node metastasis from distant metastasis can be considered in the future to better distinguish the prognosis of patients. Second, in this study, the overall lymph node metastasis rate (possibly including patients with distant metastasis) was 6.02%, and the rate of lymph node metastasis alone (not including patients with distant metastasis) was 3.42%, which was significantly different (χ2 = 110.539, p <0.001). In multivariate logistic analysis, distant metastasis was an independent risk factor for lymph node metastasis (p<0.001). Therefore, in some circumstances, distant metastasis may be partly associated with lymph node metastasis to some extent. Among the subtypes with more than 100 cases, rhabdomyosarcoma, angiosarcoma, sarcoma NOS had the top three highest percentage of lymph node metastasis. It was also noted that some subtypes, such as dermatofibrosarcoma did not have lymph node metastasis, with a lymph node metastasis rate of 0%. Other common STSs, including liposarcoma (1.47%) and fibrosarcoma (2.70%), also had a lower proportion of lymph node metastasis. Third, most of the previous studies only assessed the risk factors for lymph node metastasis based on the lymph node metastasis rate of different pathological subtypes, which had poor statistical validity, and did not evaluate the impact on lymph node metastasis based on the clinicopathologic characteristics of patients, and some studies had fewer cases (7, 10–12). One study used the NCDB database to evaluate risk factors for lymph node metastasis in 631 patients who underwent lymph node dissection, but the number of patients included was small, and the results showed a low lymph node metastasis rate in rhabdomyosarcoma, which was contrary to previous reports in the literature with little credibility (23). As a large sarcoma lymph node study, the present study comprehensively evaluated the risk factors for lymph node metastasis combined with clinicopathologic features. We found that patients with larger tumor diameters located in the head and neck, viscera, retroperitoneum, and certain specific pathological subtypes (compared with liposarcoma), such as sarcoma NOS, undifferentiated pleomorphic sarcoma, rhabdomyosarcoma, endometrial stromal sarcoma, gastrointestinal stromal tumor, synovial sarcoma, and angiosarcoma, with higher grade and distant metastasis had a higher risk of lymph node metastasis. Previous reports that assessed the lymph node metastasis risk only according to the lymph node metastasis rate showed that the lymph node metastasis risk of rhabdomyosarcoma, synovial sarcoma and angiosarcoma was high. In the present study, through a larger sample of patients and a combination of clinicopathological features, we found that in addition to rhabdomyosarcoma, synovial sarcoma and angiosarcoma, sarcoma NOS, undifferentiated pleomorphic sarcoma, endometrial stromal sarcoma, and gastrointestinal stromal tumor also had a high risk of lymph node metastasis compared with the liposarcoma.

At the same time, this study has the following shortcomings. First, the SEER database does not provide information on recurrence; therefore, we cannot assess risk factors associated with recurrence and cannot provide guidance for monitoring recurrence. Second, the metastasis information recorded in the SEER database (including distant metastasis and lymph node metastasis) was synchronous metastasis; that is, metastasis had already occurred at the time of diagnosis, and metastasis that occurred during treatment was not recorded, which may underestimate the incidence of metastasis. Third, because the treatment information included in the SEER database, including surgery, radiotherapy, chemotherapy, etc., was too biased; for example, no radiotherapy or unknown radiotherapy were both recorded as “no/unknow”, and there is no specific treatment information such as dose and regimen, so this study did not include such treatment information in order to ensure the accuracy of the results. Fourth, we lack enough data on Chinese patients to compare with the data in the SEER database.

In conclusion, in this study, we described the epidemiology of all sarcoma pathological subtypes, including incidence, age of onset, and survival differences. We also compared the proportion and survival differences between different metastatic sites, as well as survival differences between different pathological subtypes of the same metastatic site. Most importantly, we found a significant difference in survival between patients with only lymph node metastasis and those with only distant metastasis, suggesting that these two groups of patients have different prognostic factors and could be divided into separate groups in future staging to better distinguish patient prognosis. In addition, as a sarcoma lymph node study with a large number of patients included, we determined not only the proportion of lymph node metastasis for different pathological subtypes but also the risk factors for lymph node metastasis, providing guidance for the clinical treatment of STS and preoperative lymph node evaluation.

## Data Availability Statement

The raw data supporting the conclusions of this article will be made available by the authors, without undue reservation.

## Author Contributions

HL drafted the manuscript, JY designed the project. HZ, CZ, ZL, TL, TY, GZ, JY revised the manuscript. All authors approved the final manuscript.

## Funding

This work was supported by the The Science & Technology Development Fund of Tianjin Education Commission for Higher Education [2021KJ199].

## Conflict of Interest

The authors declare that the research was conducted in the absence of any commercial or financial relationships that could be construed as a potential conflict of interest.

## Publisher’s Note

All claims expressed in this article are solely those of the authors and do not necessarily represent those of their affiliated organizations, or those of the publisher, the editors and the reviewers. Any product that may be evaluated in this article, or claim that may be made by its manufacturer, is not guaranteed or endorsed by the publisher.

## Abbreviations

STS, soft tissue sarcoma; SEER, Surveillance, Epidemiology and End Results; NOS, not otherwise specified; AJCC, American Joint Commission on Cancer; APC, annual percentage change; CI, confidence interval; OR, odds ratio; mOS, median overall survival.

## References

[1] Yang Z Zheng R Zhang S Zeng H Li H Chen W. Incidence, Distribution of Histological Subtypes and Primary Sites of Soft Tissue Sarcoma in China. Cancer Biol Med (2019) 16(3):565–74. doi: 10.20892/j.issn.2095-3941.2019.0041 PMC6743618 31565485

[2] Zhang Y Wu Z Chang J Jiang W Wang Y Wang H. An Updated Incidence Trends of Soft-Tissue Sarcoma and Cancer-Specific Survival of Patients With Primary Soft-Tissue Sarcoma of Liver: A Population-Based Study. Expert Rev Gastroenterol Hepatol (2021) 15(6):689–98. doi: 10.1080/17474124.2021.1842193 33115276

[3] Trautmann F Schuler M Schmitt J. Burden of Soft-Tissue and Bone Sarcoma in Routine Care: Estimation of Incidence, Prevalence and Survival for Health Services Research. Cancer Epidemiol (2015) 39(3):440–6. doi: 10.1016/j.canep.2015.03.002 25801944

[4] Stiller CA Trama A Serraino D Rossi S Navarro C Chirlaque MD. Descriptive Epidemiology of Sarcomas in Europe: Report From the RARECARE Project. Eur J Cancer (2013) 49(3):684–95. doi: 10.1016/j.ejca.2012.09.011 23079473

[5] Youn P Milano MT Constine LS Travis LB. Long-Term Cause-Specific Mortality in Survivors of Adolescent and Young Adult Bone and Soft Tissue Sarcoma: A Population-Based Study of 28,844 Patients. Cancer (2014) 120(15):2334–42. doi: 10.1002/cncr.28733 24752471

[6] Liu X Xu J Li F Liao Z Ren Z Zhu L. Efficacy and Safety of the VEGFR2 Inhibitor Apatinib for Metastatic Soft Tissue Sarcoma: Chinese Cohort Data From NCT03121846. BioMed Pharmacother (2020) 122:109587. doi: 10.1016/j.biopha.2019.109587 31786466

[7] Johannesmeyer D Smith V Cole DJ Esnaola NF Camp ER. The Impact of Lymph Node Disease in Extremity Soft-Tissue Sarcomas: A Population-Based Analysis. Am J Surg (2013) 206(3):289–95. doi: 10.1016/j.amjsurg.2012.10.043 23806824

[8] Loya AC Prayaga AK Arora A Sundaram C Rao IS Uppin SG. Lymph Node Metastasis of Soft Tissue Tumors: A Cytomorphologic Study. Acta Cytol (2007) 51(2):153–60. doi: 10.1159/000325708 17425195

[9] Andreou D Boldt H Werner M Hamann C Pink D Tunn PU. Sentinel Node Biopsy in Soft Tissue Sarcoma Subtypes With a High Propensity for Regional Lymphatic Spread–Results of a Large Prospective Trial. Ann Oncol (2013) 24(5):1400–5. doi: 10.1093/annonc/mds650 23372051

[10] Mazeron J-J Suit HD. Lymph Nodes as Sites of Metastases From Sarcomas of Soft Tissue. Cancer (1987) 60(8):1800–8. doi: 10.1002/1097-0142(19871015)60:8<1800::aid-cncr2820600822>3.0.co;2-n 3308055

[11] Jacobs AJ Morris CD Levin AS. Synovial Sarcoma Is Not Associated With a Higher Risk of Lymph Node Metastasis Compared With Other Soft Tissue Sarcomas. Clin Orthop Relat Res (2018) 476(3):589–98. doi: 10.1007/s11999.0000000000000057 PMC6260045 29529647

[12] Fong Y Coit DG Woodruff JM Brennan MF. Lymph Node Metastasis From Soft Tissue Sarcoma in Adults. Analysis of Data From a Prospective Database of 1772 Sarcoma Patients. Ann Surg (1993) 217(1):72–7. doi: 10.1097/00000658-199301000-00012 PMC1242736 8424704

[13] Atalay C Altinok M Seref B. The Impact of Lymph Node Metastases on Survival in Extremity Soft Tissue Sarcomas. World J Surg (2007) 31(7):1433–7. doi: 10.1007/s00268-007-9078-3 17510769

[14] Doll KM Rademaker A Sosa JA. Practical Guide to Surgical Data Sets: Surveillance, Epidemiology, and End Results (SEER) Database. JAMA Surg (2018) 153(6):588–9. doi: 10.1001/jamasurg.2018.0501 29617544

[15] Cates JMM. The AJCC 8th Edition Staging System for Soft Tissue Sarcoma of the Extremities or Trunk: A Cohort Study of the SEER Database. J Natl Compr Canc Netw (2018) 16(2):144–52. doi: 10.6004/jnccn.2017.7042 29439175

[16] Fujiki M Miyamoto S Kobayashi E Sakuraba M Chuman H. Early Detection of Local Recurrence After Soft Tissue Sarcoma Resection and Flap Reconstruction. Int Orthop (2016) 40(9):1975–80. doi: 10.1007/s00264-016-3219-y 27184055

[17] Seddon B Strauss SJ Whelan J Leahy M Woll PJ Cowie F. Gemcitabine and Docetaxel Versus Doxorubicin as First-Line Treatment in Previously Untreated Advanced Unresectable or Metastatic Soft-Tissue Sarcomas (GeDDiS): A Randomised Controlled Phase 3 Trial. Lancet Oncol (2017) 18(10):1397–410. doi: 10.1016/s1470-2045(17)30622-8 PMC5622179 28882536

[18] Hengge UR Ruzicka T Tyring SK Stuschke M Roggendorf M Schwartz RA. Update on Kaposi's Sarcoma and Other HHV8 Associated Diseases. Part 1: Epidemiology, Environmental Predispositions, Clinical Manifestations, and Therapy. Lancet Infect Dis (2002) 2(5):281–92. doi: 10.1016/s1473-3099(02)00263-3 12062994

[19] Dupin N Jary A Boussouar S Syrykh C Gandjbakhche A Bergeret S. Current and Future Tools for Diagnosis of Kaposi's Sarcoma. Cancers (Basel) (2021) 13(23):5927. doi: 10.3390/cancers13235927 34885035 PMC8657166

[20] Gage MM Nagarajan N Ruck JM Canner JK Khan S Giuliano K. Sarcomas in the United States: Recent Trends and a Call for Improved Staging. Oncotarget (2019) 10(25):2462–74. doi: 10.18632/oncotarget.26809 PMC6497437 31069009

[21] Riad S Griffin AM Liberman B Blackstein ME Catton CN Kandel RA. Lymph Node Metastasis in Soft Tissue Sarcoma in an Extremity. Clin Orthop Relat Res (2004) 426):129–34. doi: 10.1097/01.blo.0000141660.05125.46 15346063

[22] Behranwala KA A'Hern R Omar AM Thomas JM. Prognosis of Lymph Node Metastasis in Soft Tissue Sarcoma. Ann Surg Oncol (2004) 11(7):714–9. doi: 10.1245/ASO.2004.04.027 15231526

[23] Maduekwe UN Herb JN Esther RJ Kim HJ Spanheimer PM. Pathologic Nodal Staging for Clinically Node Negative Soft Tissue Sarcoma of the Extremities. J Surg Oncol (2021) 123(8):1792–800. doi: 10.1002/jso.26465 PMC11022073 33751586

