# Supplementary material for: Pan-Soft Tissue Sarcoma Analysis of the Incidence, Survival, and Metastasis: A Population-Based Study Focusing on Distant Metastasis and Lymph Node Metastasis
Source: Front Oncol. 2022 Jul 7;12:890040. doi: 10.3389/fonc.2022.890040 (PMC9303001; doi:10.3389/fonc.2022.890040)
Supplement: Supplementary file 1 [file Table_1.docx]

Supplementary table1 Pathological subtypes enrolled according to ICD-O-3 code

| Histological groups | Histologic Type | ICD-O-3 | number | percentage |
| --- | --- | --- | --- | --- |
| Sarcoma, NOS |  |  | 18002 | 15.8 |
|  | Sarcoma, NOS | 8800 | 7184 | 6.3 |
|  | Spindle cell sarcoma | 8801 | 3103 | 2.7 |
|  | Giant cell sarcoma | 8802 | 4552 | 4.0 |
|  | Small cell sarcoma | 8803 | 282 | .2 |
|  | Epithelioid sarcoma | 8804 | 908 | .8 |
|  | Undifferentiated sarcoma | 8805 | 1528 | 1.3 |
|  | Desmoplastic small round cell tumor | 8806 | 445 | .4 |
| Leiomyosarcoma |  |  | 16929 | 14.9 |
|  | Leiomyosarcoma, NOS | 8890 | 15990 | 14.1 |
|  | Epithelioid leiomyosarcoma | 8891 | 484 | .4 |
|  | Angiomyosarcoma | 8894 | 42 | .0 |
|  | Myosarcoma | 8895 | 72 | .1 |
|  | Myxoid leiomyosarcoma | 8896 | 341 | .3 |
| Liposarcoma |  |  | 13564 | 11.9 |
|  | Liposarcoma, NOS | 8850 | 2162 | 1.9 |
|  | Liposarcoma, well differentiated | 8851 | 4399 | 3.9 |
|  | Myxoid liposarcoma | 8852 | 2558 | 2.2 |
|  | Round cell liposarcoma | 8853 | 223 | .2 |
|  | Pleomorphic liposarcoma | 8854 | 1000 | .9 |
|  | Mixed liposarcoma | 8855 | 371 | .3 |
|  | Fibroblastic liposarcoma | 8857 | 19 | .0 |
|  | Dedifferentiated liposarcoma | 8858 | 2832 | 2.5 |
| Gastrointestinal stromal tumour | Gastrointestinal stromal tumour | 8936 | 13024 | 11.5 |
| Kaposi sarcoma | Kaposi sarcoma | 9140 | 8838 | 7.8 |
| Dermatofibrosarcoma |  |  | 7746 | 6.8 |
|  | Dermatofibrosarcoma, NOS | 8832 | 7537 | 6.6 |
|  | Pigmented dermatofibrosarcoma protuberans | 8833 | 209 | .2 |
| Undifferentiated pleomorphic sarcoma | Undifferentiated pleomorphic sarcoma | 8830 | 7622 | 6.7 |
| Angiosarcoma | Angiosarcoma | 9120 | 4578 | 4.0 |
| Rhabdomyosarcoma |  |  | 3832 | 3.4 |
|  | Rhabdomyosarcoma, NOS | 8900 | 907 | .8 |
|  | Pleomorphic rhabdomyosarcoma | 8901 | 446 | .4 |
|  | Mixed type rhabdomyosarcoma | 8902 | 68 | .1 |
|  | Embryonal rhabdomyosarcoma | 8910 | 1315 | 1.2 |
|  | Spindle cell rhabdomyosarcoma | 8912 | 155 | .1 |
|  | Alveolar rhabdomyosarcoma | 8920 | 941 | .8 |
| Synovial sarcoma |  |  | 2783 | 2.4 |
|  | Synovial sarcoma, NOS | 9040 | 1138 | 1.0 |
|  | Synovial sarcoma, spindle cell | 9041 | 1048 | .9 |
|  | Synovial sarcoma, epithelioid cell | 9042 | 14 | .0 |
|  | Synovial sarcoma, biphasic | 9043 | 583 | .5 |
| Fibromyxosarcoma | Fibromyxosarcoma | 8811 | 2766 | 2.4 |
| Endometrial stromal sarcoma |  |  | 2723 | 2.4 |
|  | Endometrial stromal sarcoma, NOS | 8930 | 1509 | 1.3 |
|  | Endometrial stromal sarcoma, low grade | 8931 | 1214 | 1.1 |
| MPNST |  |  | 2686 | 2.4 |
|  | MPNST, NOS | 9540 | 2229 | 2.0 |
|  | Neurilemmoma, malignant | 9560 | 321 | .3 |
|  | Malignant peripheral nerve sheath tumor with  rhabdomyoblastic differentiation | 9561 | 119 | .1 |
|  | Perineurioma, malignant | 9571 | 17 | .0 |
| Primitive neuroectodermal tumor, NOS | Primitive neuroectodermal tumor, NOS | 9473 | 1120 | 1.0 |
| Fibrosarcoma |  |  | 1092 | 1.0 |
|  | Fibrosarcoma, NOS | 8810 | 977 | .9 |
|  | Fascial fibrosarcoma | 8813 | 3 | .0 |
|  | Infantile fibrosarcoma | 8814 | 112 | .1 |
| other |  |  |  |  |
|  | Solitary fibrous tumour, malignant | 8815 | 672 | .6 |
|  | Hemangiopericytoma, malignant | 9150 | 628 | .6 |
|  | Myoepithelial carcinoma | 8982 | 603 | .5 |
|  | Peripheral neuroectodermal tumor | 9364 | 507 | .4 |
|  | Stromal sarcoma, NOS | 8935 | 492 | .4 |
|  | Extraskeletal myxoid chondrosarcoma | 9231 | 459 | .4 |
|  | Myxosarcoma | 8840 | 427 | .4 |
|  | Epithelial Hemangioendothelioma | 9133 | 418 | .4 |
|  | Mixed tumour, malignant | 8940 | 400 | .4 |
|  | Rhabdoid tumour | 8963 | 392 | .3 |
|  | Clear cell sarcoma | 9044 | 305 | .3 |
|  | Alveolar soft part sarcoma | 9581 | 258 | .2 |
|  | Embryonal sarcoma | 8991 | 130 | .1 |
|  | Granular cell tumour, malignant | 9580 | 128 | .1 |
|  | Myofibroblastic sarcoma | 8825 | 125 | .1 |
|  | Hemangioendothelioma, malignant | 9130 | 120 | .1 |
|  | Phosphaturic mesenchymal tumour, malignant | 8990 | 71 | .1 |
|  | Glomus tumour, malignant | 8711 | 69 | .1 |
|  | Malignant tenosynovial giant cell tumour | 9252 | 60 | .1 |
|  | Malignant giant cell tumor of soft parts | 9251 | 57 | .1 |
|  | Ossifying fibromyxoid tumour, malignant | 8842 | 29 | .0 |
|  | Lymphangiosarcoma | 9170 | 26 | .0 |
|  | Perivascular epithelioid tumour, malignant | 8714 | 18 | .0 |
|  | Ectomesenchymoma | 8921 | 11 | .0 |
|  | Intimal sarcoma | 9137 | 5 | .0 |
| total |  |  | 113715 | 100.0 |
