# Supplementary material for: Pan-Soft Tissue Sarcoma Analysis of the Incidence, Survival, and Metastasis: A Population-Based Study Focusing on Distant Metastasis and Lymph Node Metastasis
Source: Front Oncol. 2022 Jul 7;12:890040. doi: 10.3389/fonc.2022.890040 (PMC9303001; doi:10.3389/fonc.2022.890040)
Supplement: Supplementary file 2 [file Table_2.docx]

Supplementary table2 1 -, 3 -, 5 -, 7 -, 10 -, and 15-year survival rates for all pathological subtypes

| Subtype | 1-year-survival rate (%) | 3-year-survival rate (%) | 5-year-survival rate (%) | 7-year-survival rate (%) | 10-year-survival rate (%) | 15-year-survival rate (%) |
| --- | --- | --- | --- | --- | --- | --- |
| Sarcoma, NOS | 54 | 44 | 39 | 36 | 32 | 28 |
| Leiomyosarcoma | 68 | 53 | 45 | 40 | 34 | 29 |
| Liposarcoma | 85 | 75 | 69 | 64 | 58 | 50 |
| Gastrointestinal stromal tumour | 87 | 76 | 67 | 59 | 48 | 38 |
| Dermatofibrosarcoma | 99 | 98 | 97 | 95 | 93 | 90 |
| Undifferentiated pleomorphic sarcoma | 72 | 60 | 52 | 46 | 40 | 32 |
| Angiosarcoma | 39 | 27 | 22 | 19 | 16 | 12 |
| Rhabdomyosarcoma | 64 | 52 | 48 | 47 | 47 | 46 |
| Synovial sarcoma | 79 | 64 | 57 | 54 | 51 | 48 |
| Fibromyxosarcoma | 89 | 80 | 75 | 72 | 69 | 65 |
| Endometrial stromal sarcoma | 75 | 70 | 67 | 66 | 62 | 57 |
| Malignant peripheral nerve sheath tumor | 65 | 55 | 51 | 48 | 45 | 43 |
| Primitive neuroectodermal tumor | 61 | 51 | 47 | 45 | 43 | 42 |
| Fibrosarcoma | 80 | 71 | 68 | 65 | 63 | 58 |
| Solitary fibrous tumour, malignant | 79 | 65 | 55 | 48 | 41 | 23 |
| Hemangiopericytoma, malignant | 83 | 74 | 65 | 59 | 53 | 41 |
| Myoepithelial carcinoma | 87 | 75 | 70 | 67 | 64 | 54 |
| Peripheral neuroectodermal tumor | 65 | 58 | 54 | 52 | 48 | 45 |
| Stromal sarcoma, NOS | 73 | 65 | 60 | 58 | 54 | 47 |
| Extraskeletal myxoid chondrosarcoma | 86 | 78 | 69 | 63 | 54 | 45 |
| Myxosarcoma | 82 | 74 | 68 | 60 | 56 | 48 |
| Epithelial Hemangioendothelioma | 67 | 56 | 52 | 49 | 47 | 40 |
| Mixed tumour, malignant | 79 | 64 | 58 | 52 | 46 | 42 |
| Rhabdoid tumour | 43 | 37 | 33 | 31 | 28 | 28 |
| Clear cell sarcoma | 66 | 55 | 50 | 45 | 43 | 41 |
| Alveolar soft part sarcoma | 82 | 68 | 56 | 48 | 44 | 44 |
| Embryonal sarcoma | 81 | 77 | 73 | 67 | 65 | 65 |
| Granular cell tumour, malignant | 73 | 68 | 60 | 55 | 55 | 38 |
| Myofibroblastic sarcoma | 78 | 68 | 66 | 61 | 58 | 43 |
| Hemangioendothelioma, malignant | 63 | 57 | 55 | 52 | 52 | 52 |
| Phosphaturic mesenchymal tumour, malignant | 61 | 55 | 52 | 47 | 47 | 47 |
| Glomus tumour, malignant | 86 | 79 | 76 | 73 | 73 | 73 |
| Malignant tenosynovial giant cell tumour | 89 | 89 | 86 | 82 | 82 | 82 |
| Malignant giant cell tumor of soft parts | 78 | 71 | 71 | 66 | 66 | 66 |
| Ossifying fibromyxoid tumour, malignant | 87 | 61 | 61 | 61 | - | - |
| Lymphangiosarcoma | 75 | 38 | 25 | 25 | 25 | 25 |
| Ectomesenchymoma | 60 | 60 | 60 | 60 | 60 | 60 |
