# Supplementary material for: Pan-Soft Tissue Sarcoma Analysis of the Incidence, Survival, and Metastasis: A Population-Based Study Focusing on Distant Metastasis and Lymph Node Metastasis
Source: Front Oncol. 2022 Jul 7;12:890040. doi: 10.3389/fonc.2022.890040 (PMC9303001; doi:10.3389/fonc.2022.890040)
Supplement: Supplementary file 3 [file Table_3.docx]

Supplementary table3 Bone metastases rate in different pathological subtypes

| subtype | negative | positive | total | percentage |
| --- | --- | --- | --- | --- |
| Rhabdomyosarcoma | 1272 | 200 | 1472 | 13.59% |
| Hemangioendothelioma, malignant | 33 | 4 | 37 | 10.81% |
| Alveolar soft part sarcoma | 105 | 12 | 117 | 10.26% |
| Angiosarcoma | 984 | 99 | 1083 | 9.14% |
| Epithelial Hemangioendothelioma | 130 | 12 | 142 | 8.45% |
| Peripheral neuroectodermal tumor | 152 | 14 | 166 | 8.43% |
| Clear cell sarcoma | 107 | 8 | 115 | 6.96% |
| Rhabdoid tumour | 159 | 10 | 169 | 5.92% |
| Granular cell tumour, malignant | 32 | 2 | 34 | 5.88% |
| Sarcoma, NOS | 5946 | 308 | 6254 | 4.92% |
| Primitive neuroectodermal tumor, NOS | 275 | 14 | 289 | 4.84% |
| Malignant peripheral nerve sheath tumor | 739 | 35 | 774 | 4.52% |
| Leiomyosarcoma | 5582 | 249 | 5831 | 4.27% |
| Extraskeletal myxoid chondrosarcoma | 158 | 6 | 164 | 3.66% |
| Synovial sarcoma | 1060 | 34 | 1094 | 3.11% |
| Fibrosarcoma | 250 | 7 | 257 | 2.72% |
| Hemangiopericytoma, malignant | 188 | 4 | 192 | 2.08% |
| Stromal sarcoma, NOS | 146 | 3 | 149 | 2.01% |
| Myofibroblastic sarcoma | 50 | 1 | 51 | 1.96% |
| Solitary fibrous tumour, malignant | 259 | 5 | 264 | 1.89% |
| Myxosarcoma | 159 | 3 | 162 | 1.85% |
| Mixed tumour, malignant | 107 | 2 | 109 | 1.83% |
| Myoepithelial carcinoma | 239 | 4 | 243 | 1.65% |
| Endometrial stromal sarcoma | 1021 | 14 | 1035 | 1.35% |
| Liposarcoma | 5038 | 67 | 5105 | 1.31% |
| Undifferentiated pleomorphic sarcoma | 1247 | 12 | 1259 | 0.95% |
| Fibromyxosarcoma | 1246 | 8 | 1254 | 0.64% |
| Gastrointestinal stromal tumour | 5044 | 23 | 5067 | 0.45% |
| Dermatofibrosarcoma | 2934 | 0 | 2934 | 0.00% |
| Embryonal sarcoma | 53 | 0 | 53 | 0.00% |
| Glomus tumour, malignant | 27 | 0 | 27 | 0.00% |
| Malignant tenosynovial giant cell tumour | 21 | 0 | 21 | 0.00% |
| Ossifying fibromyxoid tumour, malignant | 20 | 0 | 20 | 0.00% |
| Malignant giant cell tumor of soft parts | 16 | 0 | 16 | 0.00% |
| Perivascular epithelioid tumour, malignant | 11 | 0 | 11 | 0.00% |
| Phosphaturic mesenchymal tumour, malignant | 10 | 0 | 10 | 0.00% |
| Ectomesenchymoma | 5 | 0 | 5 | 0.00% |
| Lymphangiosarcoma | 2 | 0 | 2 | 0.00% |
