# Supplementary material for: Pan-Soft Tissue Sarcoma Analysis of the Incidence, Survival, and Metastasis: A Population-Based Study Focusing on Distant Metastasis and Lymph Node Metastasis
Source: Front Oncol. 2022 Jul 7;12:890040. doi: 10.3389/fonc.2022.890040 (PMC9303001; doi:10.3389/fonc.2022.890040)
Supplement: Supplementary file 4 [file Table_4.docx]

Supplementary table4 Brain metastases rate in different pathological subtypes

| subtype | negative | positive | total | percentage |
| --- | --- | --- | --- | --- |
| Alveolar soft part sarcoma | 110 | 7 | 117 | 5.98% |
| Rhabdoid tumour | 161 | 8 | 169 | 4.73% |
| Primitive neuroectodermal tumor, NOS | 280 | 9 | 289 | 3.11% |
| Epithelial Hemangioendothelioma | 138 | 4 | 142 | 2.82% |
| Hemangioendothelioma, malignant | 36 | 1 | 37 | 2.70% |
| Clear cell sarcoma | 112 | 3 | 115 | 2.61% |
| Angiosarcoma | 1066 | 17 | 1083 | 1.57% |
| Rhabdomyosarcoma | 1451 | 21 | 1472 | 1.43% |
| Sarcoma, NOS | 6189 | 65 | 6254 | 1.04% |
| Malignant peripheral nerve sheath tumor | 766 | 8 | 774 | 1.03% |
| Mixed tumour, malignant | 108 | 1 | 109 | 0.92% |
| Fibrosarcoma | 255 | 2 | 257 | 0.78% |
| Stromal sarcoma, NOS | 148 | 1 | 149 | 0.67% |
| Extraskeletal myxoid chondrosarcoma | 163 | 1 | 164 | 0.61% |
| Leiomyosarcoma | 5800 | 31 | 5831 | 0.53% |
| Endometrial stromal sarcoma | 1030 | 5 | 1035 | 0.48% |
| Synovial sarcoma | 1089 | 5 | 1094 | 0.46% |
| Myoepithelial carcinoma | 242 | 1 | 243 | 0.41% |
| Solitary fibrous tumour, malignant | 263 | 1 | 264 | 0.38% |
| Undifferentiated pleomorphic sarcoma | 1256 | 3 | 1259 | 0.24% |
| Liposarcoma | 5097 | 8 | 5105 | 0.16% |
| Gastrointestinal stromal tumour | 5064 | 3 | 5067 | 0.06% |
| Dermatofibrosarcoma | 2934 | 0 | 2934 | 0.00% |
| Fibromyxosarcoma | 1254 | 0 | 1254 | 0.00% |
| Hemangiopericytoma, malignant | 192 | 0 | 192 | 0.00% |
| Peripheral neuroectodermal tumor | 166 | 0 | 166 | 0.00% |
| Myxosarcoma | 162 | 0 | 162 | 0.00% |
| Embryonal sarcoma | 53 | 0 | 53 | 0.00% |
| Myofibroblastic sarcoma | 51 | 0 | 51 | 0.00% |
| Granular cell tumour, malignant | 34 | 0 | 34 | 0.00% |
| Glomus tumour, malignant | 27 | 0 | 27 | 0.00% |
| Malignant tenosynovial giant cell tumour | 21 | 0 | 21 | 0.00% |
| Ossifying fibromyxoid tumour, malignant | 20 | 0 | 20 | 0.00% |
| Malignant giant cell tumor of soft parts | 16 | 0 | 16 | 0.00% |
| Perivascular epithelioid tumour, malignant | 11 | 0 | 11 | 0.00% |
| Phosphaturic mesenchymal tumour, malignant | 10 | 0 | 10 | 0.00% |
| Ectomesenchymoma | 5 | 0 | 5 | 0.00% |
| Lymphangiosarcoma | 2 | 0 | 2 | 0.00% |
