# Supplementary material for: Pan-Soft Tissue Sarcoma Analysis of the Incidence, Survival, and Metastasis: A Population-Based Study Focusing on Distant Metastasis and Lymph Node Metastasis
Source: Front Oncol. 2022 Jul 7;12:890040. doi: 10.3389/fonc.2022.890040 (PMC9303001; doi:10.3389/fonc.2022.890040)
Supplement: Supplementary file 5 [file Table_5.docx]

Supplementary table5 Liver metastases rate in different pathological subtypes

| Subtype | negative | positive | total | percentage |
| --- | --- | --- | --- | --- |
| Epithelial Hemangioendothelioma | 124 | 18 | 142 | 12.68% |
| Gastrointestinal stromal tumour | 4520 | 547 | 5067 | 10.80% |
| Leiomyosarcoma | 5410 | 421 | 5831 | 7.22% |
| Rhabdoid tumour | 157 | 12 | 169 | 7.10% |
| Angiosarcoma | 1014 | 69 | 1083 | 6.37% |
| Granular cell tumour, malignant | 32 | 2 | 34 | 5.88% |
| Hemangioendothelioma, malignant | 35 | 2 | 37 | 5.41% |
| Stromal sarcoma, NOS | 141 | 8 | 149 | 5.37% |
| Peripheral neuroectodermal tumor | 158 | 8 | 166 | 4.82% |
| Sarcoma, NOS | 5974 | 280 | 6254 | 4.48% |
| Glomus tumour, malignant | 26 | 1 | 27 | 3.70% |
| Clear cell sarcoma | 111 | 4 | 115 | 3.48% |
| Alveolar soft part sarcoma | 113 | 4 | 117 | 3.42% |
| Primitive neuroectodermal tumor, NOS | 280 | 9 | 289 | 3.11% |
| Rhabdomyosarcoma | 1427 | 45 | 1472 | 3.06% |
| Hemangiopericytoma, malignant | 187 | 5 | 192 | 2.60% |
| Malignant peripheral nerve sheath tumor | 756 | 18 | 774 | 2.33% |
| Solitary fibrous tumour, malignant | 258 | 6 | 264 | 2.27% |
| Endometrial stromal sarcoma | 1014 | 21 | 1035 | 2.03% |
| Myxosarcoma | 159 | 3 | 162 | 1.85% |
| Mixed tumour, malignant | 107 | 2 | 109 | 1.83% |
| Synovial sarcoma | 1080 | 14 | 1094 | 1.28% |
| Fibrosarcoma | 254 | 3 | 257 | 1.17% |
| Liposarcoma | 5050 | 55 | 5105 | 1.08% |
| Undifferentiated pleomorphic sarcoma | 1250 | 9 | 1259 | 0.71% |
| Extraskeletal myxoid chondrosarcoma | 163 | 1 | 164 | 0.61% |
| Myoepithelial carcinoma | 242 | 1 | 243 | 0.41% |
| Fibromyxosarcoma | 1252 | 2 | 1254 | 0.16% |
| Dermatofibrosarcoma | 2933 | 1 | 2934 | 0.03% |
| Embryonal sarcoma | 53 | 0 | 53 | 0.00% |
| Myofibroblastic sarcoma | 51 | 0 | 51 | 0.00% |
| Malignant tenosynovial giant cell tumour | 21 | 0 | 21 | 0.00% |
| Ossifying fibromyxoid tumour, malignant | 20 | 0 | 20 | 0.00% |
| Malignant giant cell tumor of soft parts | 16 | 0 | 16 | 0.00% |
| Perivascular epithelioid tumour, malignant | 11 | 0 | 11 | 0.00% |
| Phosphaturic mesenchymal tumour, malignant | 10 | 0 | 10 | 0.00% |
| Ectomesenchymoma | 5 | 0 | 5 | 0.00% |
| Lymphangiosarcoma | 2 | 0 | 2 | 0.00% |
