# Supplementary material for: Pan-Soft Tissue Sarcoma Analysis of the Incidence, Survival, and Metastasis: A Population-Based Study Focusing on Distant Metastasis and Lymph Node Metastasis
Source: Front Oncol. 2022 Jul 7;12:890040. doi: 10.3389/fonc.2022.890040 (PMC9303001; doi:10.3389/fonc.2022.890040)
Supplement: Supplementary file 6 [file Table_6.docx]

Supplementary table6 Lung metastases rate in different pathological subtypes

| Subtype | negative | positive | total | percentage |
| --- | --- | --- | --- | --- |
| Alveolar soft part sarcoma | 62 | 55 | 117 | 47.01% |
| Epithelial Hemangioendothelioma | 103 | 39 | 142 | 27.46% |
| Phosphaturic mesenchymal tumour, malignant | 8 | 2 | 10 | 20.00% |
| Rhabdoid tumour | 136 | 33 | 169 | 19.53% |
| Hemangioendothelioma, malignant | 31 | 6 | 37 | 16.22% |
| Rhabdomyosarcoma | 1254 | 218 | 1472 | 14.81% |
| Leiomyosarcoma | 5007 | 824 | 5831 | 14.13% |
| Extraskeletal myxoid chondrosarcoma | 141 | 23 | 164 | 14.02% |
| Angiosarcoma | 937 | 146 | 1083 | 13.48% |
| Peripheral neuroectodermal tumor | 144 | 22 | 166 | 13.25% |
| Sarcoma, NOS | 5484 | 770 | 6254 | 12.31% |
| Synovial sarcoma | 972 | 122 | 1094 | 11.15% |
| Endometrial stromal sarcoma | 937 | 98 | 1035 | 9.47% |
| Embryonal sarcoma | 48 | 5 | 53 | 9.43% |
| Perivascular epithelioid tumour, malignant | 10 | 1 | 11 | 9.09% |
| Clear cell sarcoma | 106 | 9 | 115 | 7.83% |
| Malignant peripheral nerve sheath tumor | 716 | 58 | 774 | 7.49% |
| Malignant giant cell tumor of soft parts | 15 | 1 | 16 | 6.25% |
| Granular cell tumour, malignant | 32 | 2 | 34 | 5.88% |
| Mixed tumour, malignant | 103 | 6 | 109 | 5.50% |
| Stromal sarcoma, NOS | 141 | 8 | 149 | 5.37% |
| Solitary fibrous tumour, malignant | 250 | 14 | 264 | 5.30% |
| Primitive neuroectodermal tumor, NOS | 274 | 15 | 289 | 5.19% |
| Malignant tenosynovial giant cell tumour | 20 | 1 | 21 | 4.76% |
| Fibrosarcoma | 245 | 12 | 257 | 4.67% |
| Undifferentiated pleomorphic sarcoma | 1207 | 52 | 1259 | 4.13% |
| Hemangiopericytoma, malignant | 186 | 6 | 192 | 3.13% |
| Myxosarcoma | 158 | 4 | 162 | 2.47% |
| Liposarcoma | 4995 | 110 | 5105 | 2.15% |
| Fibromyxosarcoma | 1227 | 27 | 1254 | 2.15% |
| Myoepithelial carcinoma | 238 | 5 | 243 | 2.06% |
| Myofibroblastic sarcoma | 50 | 1 | 51 | 1.96% |
| Gastrointestinal stromal tumour | 5021 | 46 | 5067 | 0.91% |
| Dermatofibrosarcoma | 2931 | 3 | 2934 | 0.10% |
| Glomus tumour, malignant | 27 | 0 | 27 | 0.00% |
| Ossifying fibromyxoid tumour, malignant | 20 | 0 | 20 | 0.00% |
| Ectomesenchymoma | 5 | 0 | 5 | 0.00% |
| Lymphangiosarcoma | 2 | 0 | 2 | 0.00% |
