# Supplementary material for: Pan-Soft Tissue Sarcoma Analysis of the Incidence, Survival, and Metastasis: A Population-Based Study Focusing on Distant Metastasis and Lymph Node Metastasis
Source: Front Oncol. 2022 Jul 7;12:890040. doi: 10.3389/fonc.2022.890040 (PMC9303001; doi:10.3389/fonc.2022.890040)
Supplement: Supplementary file 7 [file Table_7.docx]

Supplementary table7 Lymph node metastasis rate in pathological subtypes with patients <100 cases

| subtype | Negative | positive | total | percentage |
| --- | --- | --- | --- | --- |
| Phosphaturic mesenchymal tumour, malignant | 4 | 3 | 7 | 42.86% |
| Ectomesenchymoma | 2 | 1 | 3 | 33.33% |
| Mixed tumour, malignant | 47 | 23 | 70 | 32.86% |
| Clear cell sarcoma | 56 | 16 | 72 | 22.22% |
| Peripheral neuroectodermal tumor | 72 | 14 | 86 | 16.28% |
| Primitive neuroectodermal tumor | 38 | 7 | 45 | 15.56% |
| Rhabdoid tumour | 34 | 5 | 39 | 12.82% |
| Alveolar soft part sarcoma | 58 | 8 | 66 | 12.12% |
| Stromal sarcoma, NOS | 78 | 9 | 87 | 10.34% |
| Epithelial Hemangioendothelioma | 29 | 3 | 32 | 9.38% |
| Granular cell tumour, malignant | 11 | 1 | 12 | 8.33% |
| Extraskeletal myxoid chondrosarcoma | 92 | 7 | 99 | 7.07% |
| Myofibroblastic sarcoma | 27 | 1 | 28 | 3.57% |
| Myxosarcoma | 87 | 3 | 90 | 3.33% |
| Hemangiopericytoma, malignant | 59 | 1 | 60 | 1.67% |
| Solitary fibrous tumour, malignant | 85 | 0 | 85 | 0.00% |
| Ossifying fibromyxoid tumour, malignant | 10 | 0 | 10 | 0.00% |
| Malignant tenosynovial giant cell tumour | 10 | 0 | 10 | 0.00% |
| Hemangioendothelioma, malignant | 8 | 0 | 8 | 0.00% |
| Malignant giant cell tumor of soft parts | 7 | 0 | 7 | 0.00% |
| Embryonal sarcoma | 4 | 0 | 4 | 0.00% |
| Lymphangiosarcoma | 1 | 0 | 1 | 0.00% |
